# Neurocognitive Artificial Neural Network Models Are Superior to Linear Models at Accounting for Dimensional Psychopathology

**DOI:** 10.3390/brainsci12081060

**Published:** 2022-08-10

**Authors:** Darren Haywood, Frank D. Baughman, Barbara A. Mullan, Karen R. Heslop

**Affiliations:** 1 St. Vincent’s Hospital Melbourne, Mental Health, Fitzroy, VIC 3065, Australia; 2 School of Population Health, Curtin University, Bentley, WA 6102, Australia; 3 EnAble Institute, Curtin University, Bentley, WA 6102, Australia; 4 Curtin School of Nursing, Curtin University, Bentley, WA 6102, Australia

**Keywords:** neurocognition, psychopathology, mental health, dimensional, CFA, artificial neural network, modelling, *p*-factor, internalising, externalising

## Abstract

In recent years, there has been debate about the optimal conceptualisation of psychopathology. Structural models of psychopathology have been developed to counter issues, including comorbidity and poor diagnostic stability prevalent within the traditional nosological approach. Regardless of the conceptualisation of psychological dysfunction, deficits in neurocognitive abilities have been claimed to be an aetiological feature of psychopathology. Explorations of the association between neurocognition and psychopathology have typically taken a linear approach, overlooking the potential interactive dynamics of neurocognitive abilities. Previously, we proposed a multidimensional hypothesis, where within-person interactions between neurocognitive domains are fundamental to understanding the role of neurocognition within psychopathology. In this study, we used previously collected psychopathology data for 400 participants on psychopathological symptoms, substance use, and performance on eight neurocognitive tasks and compared the predictive accuracy of linear models to artificial neural network models. The artificial neural network models were significantly more accurate than the traditional linear models at predicting actual (a) lower-level and (b) high-level dimensional psychopathology. These results provide support for the multidimensional hypothesis: that the study of non-linear interactions and compensatory neurocognitive profiles are integral to understanding the functional associations between neurocognition and of psychopathology.

## 1. Introduction

It has been contended that deficits in neurocognitive abilities are an aetiological feature of psychopathology [1,2]. Not only do those with psychopathology typically have neurocognitive deficits, but these deficits in neurocognitive performance are seen to precede the development of psychopathology [2]. However, few, if any, deficits to underlying neurocognitive abilities appear to be deterministic in the study of psychopathology. Thus, the search for one-to-one correspondence between deficits and disorders has yielded little knowledge that can be constantly applied. Instead, evidence suggests that the associations between neurocognitive abilities and psychopathology are extensively heterogenous [3,4,5,6,7,8,9]. For example, previous research has found that for people with bipolar disorder approximately 22% displayed deficits in three to four neurocognitive components, 40% showed deficits in one or two components, and 38% did not display any deficits [9]. Of note is the fact that no consistent deficit could be isolated, in any single neurocognitive component. Multi-disorder research corroborates these findings as there is no evidence for specific, single neurocognitive deficits that reliably discriminate disorders [4].

One possible explanation for the extensive heterogeneity of the associations between neurocognitive abilities and psychopathology may stem from the use of traditional nosological approaches to diagnosis. Traditional approaches to diagnosis, using tools such as the DSM, have resulted in extensive comorbidity, and poor diagnostic stability [10,11]. The high levels of comorbidity and poor diagnostic stability, along with emerging aetiological evidence [12,13,14,15], suggests that psychopathology may not be best represented as discrete diagnostic categories [16,17], and if this is the case, finding associations between any particular neurocognitive component and a specific diagnostic category is unlikely. In recent years, the issues with the traditional nosological approach to diagnosis has led to the development of a range of dimensional, statistical models of psychopathology [18,19,20]. These models of psychopathology do not categorise disorder, but rather represent symptoms dimensionally on a collection of higher-order statistically derived components of psychopathology. The models of psychopathology that have gained most interest are the correlated factors model, the bifactor model, and the single-factor model. The correlated factors model contains a range of lower-level symptom indicators, such as depression, anxiety, and hostility, and two or more higher-level correlated dimensional factors, such as internalising and externalising [21]. The bifactor model is similar in structure, however it includes a single factor, named the *p*-factor, at the highest level of the structure that also receives its loadings from the lower-level symptom indicators [18]. The single-factor model contains the same lower-level symptom indicators, but only the higher-level *p*-factor [18]. Previously, we suggested that by using these dimensional statistical models it may be possible to find clear specific associations between different neurocognitive abilities and the factors of psychopathology that discriminate the factors [22]. However, more recently, only a common deficit in speed of processing was found to be related to higher scores of internalising, externalising and *p*, providing evidence that there may not be discrete neurocognitive associations among the factors [23].

The challenges faced with isolating neurocognitive deficits associated with specific disorders, and the issues concerning the heterogeneity of behavioural symptoms in disorders, point to the need for approaches that can examine the effects of dynamical interplay in the underlying processes. Towards this end, we recently used computational models of the Wisconsin Card Sorting Task (WCST) to explore an alternative conceptualisation of the relation between neurocognitive abilities and psychopathology, termed the multidimensional hypothesis [8,24]. Ultimately, we claimed that to understand the functional associations between neurocognition and psychopathology consideration of the non-linear interactions between neurocognitive components is of critical importance. This conceptualisation was inspired by the notion of Multiple Realisation from the study of philosophy of mind; referring to the idea that any given state may be equally determined, or realised, by a number of different causes [25]. Rather than attempting to test which single process or ability related most to a given disorder, we tested the combined effects of various profiles of neurocognitive abilities on models’ performance on the WCST. The results of this work revealed that a range of manipulations to the processes pertaining to neurocognitive abilities of updating, shifting, and inhibition were equivalent in simulating performance on the WCST in people with schizophrenia, their healthy-first degree relatives, and controls. These findings, we argue, highlight the advantages of using multidimensional approaches in the study of psychopathology [24].

Artificial neural networks (ANN) have been successful used across levels, from psychological to genetic to help understand psychopathological and behavioural phenomena [26,27,28,29,30,31,32,33] and potentially provides an even richer methodology for studying the relations between neurocognitive abilities and psychopathology. For example, in a standard 3-layer feed-forward network, for which a problem may be specified and for which the desired outcome is known, input units are provided with a representation of the problem and the output of each unit is fed forward to all units it is connected to within a hidden layer (comprised of a number of processing units). The hidden units in turn feed forward to the output layer that represents the solution. Throughout the model, each connection partially determines (via its strength of connection, or weight) the final value, and the degree of error in the model’s solution is then used to alter weights within the model with the goal of achieving a more accurate outcome on the next cycle. Traditionally, research in the domain of neurocognition and psychopathology has relied on linear explanations, often using popular correlational techniques. For example, multiple linear regression allows one to determine the unique and common contributions for any number of independent variables on an outcome. Whilst multiple linear regression is particularly accessible and easy to perform, using any of a variety of modern statistical analyses packages, it allows only for additive combinations of a linear form [34]. The issue with this is that for many psychological phenomena, it appears that rather than contributing linearly to such outcomes, that instead factors interact in more dynamic ways in producing effects.

Towards the other end of the complexity spectrum, in terms of analytical techniques, are machine learning techniques, or artificial neural networks (ANN). The potential advantage of such approaches is that they allow highly complex, non-linear patterns of relations to be found between any number of variables and an outcome. However, studies examining the difference between standard analytical techniques, such as MLR, and machine learning approaches are lacking. Knowing what the potential benefits are, of one approach over another, offers clear advantages for elucidating the true role factors play in influencing specific outcomes, and the degree to which multidimensionality holds. The central objective of this study is to compare multiple linear regression models (MLR) to artificial neural network models (ANN), in order to determine the degree to which each are able to predict specific psychopathological outcomes. In each instance, to facilitate comparison, the models we develop represents the more accessible of techniques that exist with the respective approaches.

## 2. Aims and Hypotheses

The aim of this research is to compare the accuracy of linear models versus non-linear artificial neural network models with regard to how well they each predict (a) lower-level and (b) higher-level psychopathology.

**Hypothesis** **1.** *The average correlations between the actual lower-level psychopathology scores and the models’ predicted psychopathology scores will be significantly stronger for the ANN model when compared to the linear model.*

**Hypothesis** **2.** *The correlations between actual and model predicted (a) internalising, (b) externalising, and (c) general psychopathology (the p-factor) scores will be significantly stronger for the ANN models when compared to the linear models.*

## 3. Materials and Methods

### 3.1. Participants

In a large-scale study [23], 425 people from a representative community sample from the USA were recruited through Prolific [35]. Participants completed a demographics and clinical characteristics survey [36], a substance use measure [37], and eight neurocognitive tasks. After data cleaning 400 participants were retained. The mean age of the sample was 44.47 (SD = 16.35), 51.5% were female, and 28.5% reported having a previous or current mental health diagnosis. The detailed demographic and clinical characteristics of the sample can be found in Haywood, Baughman, Mullan and Heslop [23].

### 3.2. Procedure

After providing consent, participants completed the demographic and clinical characteristics questions, and then completed measures on substance use, mental health symptomology, and then each of the eight neurocognitive tasks see [23] for further information. This research was approved by the Curtin Human Research Ethics Committee (HRE2021-0105).

### 3.3. Measures

In this study, we used a subset of variables collected in the larger study [23]. We used structural models of psychopathology developed in the larger study derived from data collected using the Alcohol, Smoking and Substance Involvement Screening Test (ASSIST) V3.1 [37], and the Brief Symptom Inventory (BSI-53) 53 item [36]. The ASSIST is the gold-standard measure for substance involvement across tobacco products, alcoholic beverages, cannabis, cocaine, amphetamine-type stimulants, inhalants, sedatives or sleeping pills, hallucinogens, opioids, and other substances [37]. The BSI is a 53-item psychiatric symptom measure that assesses degree of distress associated with a wide-range of psychiatric symptoms over the previous seven days [36].

Data from eight computerised neurocognitive tasks were also collected. To measure working memory we used the Digit Span task, and a visual array task based on Cowen [38]. To measure shifting we used the Shape-Number task, based on the Letter-Number task [39], and the Inferring Relevance Task [40]. To measure inhibition, we used a computerised version of the Stroop task [41] and the Go/NoGo task [42]. Lastly, to measure speed of processing we used the Simple Reaction Time task, and the Inspection Time (IT) task [43]. The Rate-Corrected Score (RCS) method was used for tasks that required both speed and accuracy to measure performance. Haywood, Baughman, Mullan and Heslop [23] provides further detail on the tasks used and the metrics assessed.

### 3.4. Analysis

**Figure 1:**
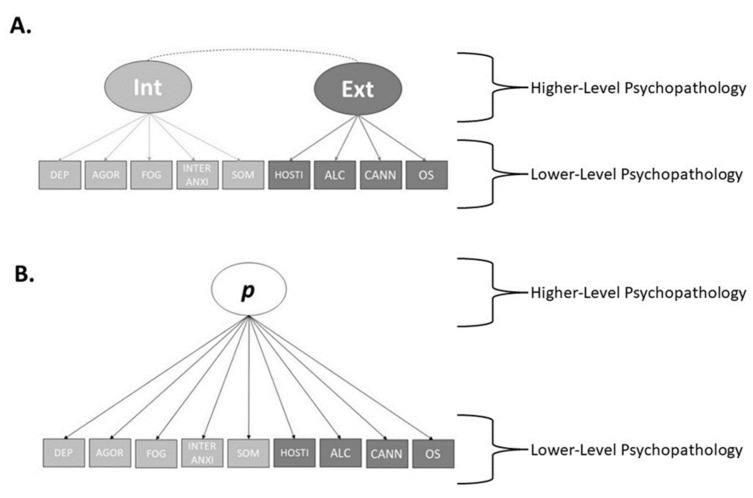
Correlated Factors Model and the Single Factor Model. **Note.** Adapted from Haywood, Baughman, Mullan and Heslop [23], final structural models of psychopathology used in this research. Pictured is the Correlated Factors Model (**A**) and Bifactor Model (**B**). DEP = Depression. AGOR = Agoraphobia, FOG = Mental Fog, INTER ANXI = Interpersonal Anxiety. SOM = Somatisation, HOSTI = Hostility. ALC = Alcohol. CANN = Cannabis. OS = Other Substances.

In this study, we used structural models of psychopathology that had previously been developed [23]. These structural models were developed and tested though confirmatory factor analysis in line with structural and hierarchical conceptual interpretations of psychopathology [18]. These models used a six-factor BSI model [44], with the six domains being Depression, Agoraphobia, Hostility, Mental Fog, Interpersonal Anxiety, and Somatisation, and three domains of substance use derived from the ASSIST V3.1, namely alcohol use, cannabis use, and other substance use. These nine domains were included as ‘lower-level’ indicators in our structural models. Regarding the structural models, we used the correlated factors model, with internalising and externalising specific factors, and the single factors model, developed previously [23]. However, the bifactor model was not used as it had a Heywood case (a variable with a negative variance estimate). The models included the BSI domains, derived from the Schwannauer and Chetwynd [44] factor structure, and the ASSIST components as the observed variables see [23] for further detail. All models were developed and tested in RStudio using the MLR estimator with robust test statistics, and the final models were chosen from the alternatives based on a combination of model fit, factor loadings, and conceptual interpretation see [23]. The final correlated-factors model and the single factor model are depicted in Figure 1.

Factor scores for internalising, externalising, and the *p*-factor were extracted for each participant. These scores were the ‘higher-level’ psychopathology variables that the linear and ANNs models were to predict to test hypothesis 2. Further, we used the scores for each of the six BSI variables, and the three ASSIST variables, as the ‘lower-level’ psychopathology scores that the two types of models were to predict in order to test hypothesis 1.

#### 3.4.1. Linear Models

Multivariate multiple regression analyses were used as the linear method to predict psychopathology from neurocognitive abilities. The models included the eight neurocognitive tasks, as well as age and gender as predictors. The outcome variables for the lower-level model were the six BSI domains; Depression, Agoraphobia, Hostility, Mental Fog, Interpersonal Anxiety, and Somatisation, and the three ASSIST variables; Alcohol, Cannabis, and other drug use. The higher-level psychopathology model included the same predictors, but the outcome variables were internalising, externalising, and *p*-factor scores.

#### 3.4.2. Artificial Neural Network Models

We developed two ANN models, one for lower-level psychopathology, and one for higher-level psychopathology. Both models were 3-layer feedforward connectionist networks consisting of an input layer of 10 units (representing age, gender, and performance on each of the eight cognitive tasks) a hidden layer of 10 units, and an output layer of either 9 units (lower-level psychopathology model) or 3 units (higher-level psychopathology model). In the lower-level model, the output layer comprised of 9 units, representing depression, agoraphobia, mental fog, interpersonal anxiety, somatisation, hostility, alcohol, cannabis, and other substances, while in the higher-level model the output layer consisted of 3 units, representing internalising, externalising and the *p*-factor. We used sigmoidal activation functions for units and the model was trained randomly, with replacement, on 100 of the 400 cases using back-propagation for 1000 epochs, with a learning rate of 0.03, and with the initial weights for all units randomised between ±0.5. The model was tested against the full set of 400 cases. To safeguard against possible under, or over-fitting our data, we examined the effect of varying the learning rate (0.01 to 0.5), and the number of hidden units (5, 10, 15, 20). These manipulations to the model’s parameters did not alter the outcome or pattern of results, although greater differentiation was noted for some extremes. For example, by the end of training, a higher learning rate (0.5) had little effect in reducing error in the model with 5 units in the hidden layer. In contrast, in those models with greater than 5 units in the hidden layer (i.e., 10, 15, 20) by the end of training, error was considerably smaller.

We did not explore the effects of using different activation function nor did we examine the effect of increasing the number of hidden layers in the model. These variations potentially may be of interest to us for future work. However, overall, and given the purpose of this study, to compare linear models to ANN models, the model described here offers a useful starting framework. The models were developed in MatLab.

**Figure 2:**
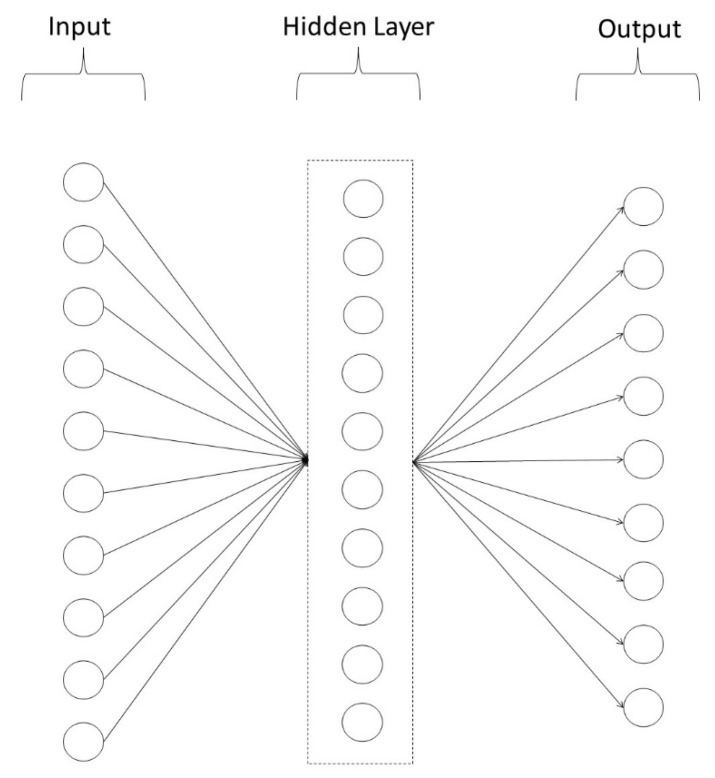
Lower-level Psychopathology Artificial Neural Network Model. **Note**. Depiction of the lower-level psychopathology artificial neural network model used in this research. The first layer of the model contains 10 input units consisting of age, gender, and the eight neurocognitive tasks. The second layer is the hidden layer consisting of 10 units. The final layer is the output layer consisting of nine output units, namely depression, agoraphobia, mental fog, interpersonal anxiety, somatisation, hostility, alcohol, cannabis, and other substances.

**Figure 3:**
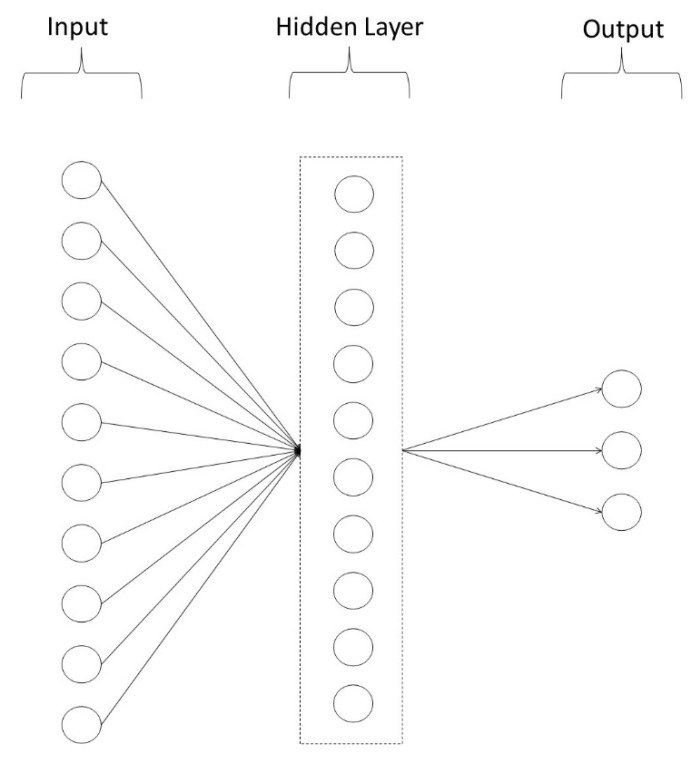
Higher-level Psychopathology Artificial Neural Network Model. **Note.** Depiction of the higher-level psychopathology artificial neural network model used in this research. The first layer of the model contains 10 input units consisting of age, gender, and the eight neurocognitive tasks. The second layer is the hidden layer consisting of 10 units. The final layer is the output layer consisting of three output units, namely internalising, externalising, and the *p*-factor.

Figure 2 depicts the lower-level psychopathology ANN model, while Figure 3 depicts the higher-level psychopathology ANN model.

#### 3.4.3. Model Comparison

The predictive accuracy of the linear models and the ANN models was assessed by statistically comparing the correlations between the respective models’ predicted outcome variable scores, and the actual outcome variable scores. The correlations between the models’ predicted and actual scores for Depression, Agoraphobia, Hostility, Mental Fog, Interpersonal Anxiety, Somatisation, Alcohol, Cannabis, and Other Drugs were averaged to provide an overall indication of the predictive accuracy of the lower-level psychopathology models. The overall correlation for the linear and the ANN was compared using the Daniel Soper calculator [45], that applies a Fisher transformation [46] to compare two correlations. Similarly, the correlations between the predicted and actual (a) internalising, (b) externalising, and (c) *p*-factor scores were statistically compared for the linear and the ANN model. Superior predictive accuracy of the ANN over the linear models at both the lower-level (BSI and ASSIST variables) and higher-level of psychopathology (internalising, externalising and *p*-factor), would evidence the existence of non-linear interactive relationships between the predictors (neurocognition, age, and gender) and the outcomes (psychopathology) [47].

## 4. Results

### 4.1. Linear Models

#### 4.1.1. Lower-Level Psychopathology

Regarding lower-level psychopathology, the linear model with the predictor variables of the eight neurocognitive variables and age and gender were able to account for a significant amount of variance in each of the nine symptom domains. The model accounted for 18.9% of depression (F (10, 389) = 9.08, *p* < 0.001, R2 = 0.189), 10.9% of agoraphobia (F (10, 389) = 4.77, *p* < 0.001, R2 = 0.109), 17.1% of hostility (F (10, 389) = 8.03, *p* < 0.001, R2 = 0.171), 22.7% of Mental Fog (F (10, 389) = 11.40, *p* < 0.001, R2 = 0.227), 20.1% of interpersonal anxiety (F (10, 389) = 9.80, *p* < 0.001, R2 = 0.201), 22.0% of somatisation (F (10, 389) = 10.98, *p* < 0.001, R2 = 0.220), 7.2% of alcohol use (F (10, 389) = 3.00, *p* = 0.001, R2 = 0.072), 5.5% of cannabis use (F (10, 389) = 2.28, *p* = 0.013, R2 = 0.055), and 5.9% of other substance use (F (10, 389) = 2.43, *p* = 0.008, R2 = 0.238). Table 1 provides the utility of the individual predictors in the model.

The neurocognitive performance tasks failed to account for any unique variance in depression, agoraphobia, and mental fog. The speed of processing tasks provided unique predictive utility for the remaining six lower-level psychopathology domains. The working memory tasks were also able to account for unique variance in other substance use. No other neurocognitive tasks offered unique predictive utility for any of the symptom domains.

#### 4.1.2. Higher-Level Psychopathology

As reported in Haywood, Baughman, Mullan and Heslop [23], our multivariate multiple regression analyses revealed that our eight neurocognitive tasks in addition to age and gender accounted for a significant amount of variance in each internalising, externalising and the *p*-factor. The model accounted for 23.8% of the variance in internalising (F(10, 389) = 12.17, *p* < 0.001, R2 = 0.238), 15.6% of the variance in externalising (F(10, 389) = 8.37, *p* < 0.001, R2 = 0.156), and 23.6% of the variance in the *p*-factor (F(10, 389) = 12.05, *p* < 0.001, R2 = 0.236). Table 2 provides the results of the regression analyses as reported in Haywood, Baughman, Mullan and Heslop [23].

Only the neurocognitive tasks measuring speed of processing accounted for significant unique variance in higher-level psychopathology. Simple reaction time and Inspection Time were significant predictors of internalising and the *p*-factor, while simple reaction time was the sole significant predictor of externalising, bar age and gender. Tasks that measured working memory, shifting, or inhibition did not provide any unique predictive utility for the higher-level psychopathology factors. For further detail of these results see Haywood, Baughman, Mullan and Heslop [23].

### 4.2. Artificial Neural Network Models

#### Lower-Level Psychopathology

**Figure 4:**
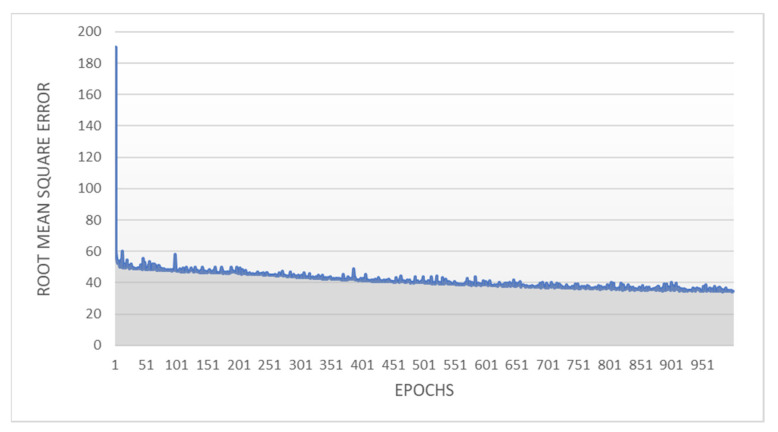
Root Mean Squared Error to Epochs for the Lower-level Psychopathology Artificial Neural Network Model. **Note.** Depiction of the accuracy and learning rate of the higher-level psychopathology artificial neural network model used in this research. The grey area under the blue line represents the summed squared error of the model that after particular number of epochs.

Over the 1000 epochs of the basic backwards propagation, the lower-level psychopathology ANN model provided a final summed squared error of 34.76 and root mean squared error (RMSE) of 0.29. The model performed well with the relatively small number of hidden units and a single hidden unit layer and learned very efficiently. For example, the summed squared error dropped from 190.27 following the first epoch to just 53.99 following the sixth epoch, and then learned steadily to end at a summed squared error of 34.76 on the 1000th epoch. The summed squared error to epochs for the lower-level psychopathology ANN are depicted in Figure 4.

### 4.3. Model Comparison

#### 4.3.1. Lower-Level Psychopathology

The bivariate correlations between each lower-level psychopathology domain scores and the linear model and ANN model predicted scores are presented in Table 3. To allow easier comparisons to be made between linear and ANN approaches, Table 3 shows the results for linear and ANN models next to one another. For instance, the table shows the correlation between the observed depression scores and that predicted by the linear model (LM-Dep) is r = 0.435, versus r = 0.648 in the neural network model (ANN-Dep).

#### 4.3.2. Higher-Level Psychopathology

**Figure 5:**
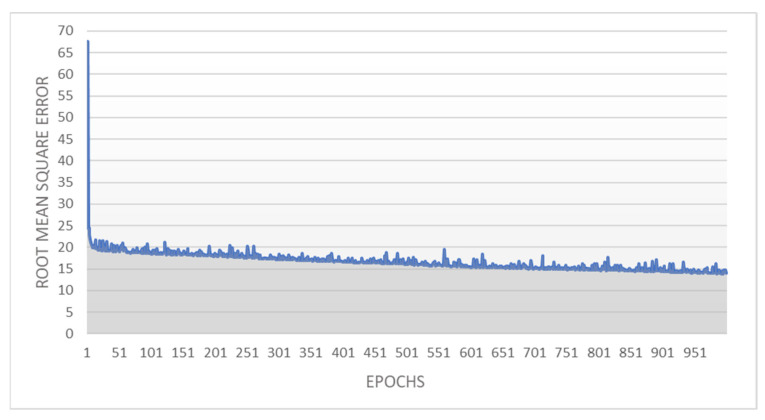
Root Mean Squared Error to Epochs for the Higher-Level Psychopathology Artificial Neural Network Model. **Note.** Depiction of the accuracy and learning rate of the higher-level psychopathology artificial neural network model used in this research. The grey area under the blue line represents the summed squared error of the model that after particular number of epochs.

Over the 1000 epochs the higher-level psychopathology ANN model provided a final summed squared error of 14.02 and a RMSE of 0.19. The higher-level psychopathology ANN performed better than the lower-level psychopathology ANN model (had a lower RMSE), however this may be attributed to the lower-level model having twice the number of output units. Again, even although the model was basic with a relatively small number of hidden units, and a single hidden unit layer, it learned efficiently. The summed squared error dropped from 67.55 following the first epoch to just 21.71 following the fifth epoch and learned progressively to end at a summed squared error of 14.02 on the 1000th epoch. The summed squared error to epochs for the higher-level psychopathology ANN are depicted in Figure 5.

For each of the nine lower-level symptom domains the predicted values of the ANN model had a stronger correlation with the actual values when compared to the linear model. The correlations between the linear model’s predicted values and the actual symptom values ranged between 0.243 and 0.476, while the correlations between the predicted values of the ANN model and the actual symptom values ranged between 0.338 and 0.711. The average correlation between the linear model’s predicted values and the actual values was 0.369, while the average correlation between the ANN’s predicted values and the actual values was 0.587. The difference between the linear and ANN models’ average correlations with the actual values amongst the lower-level psychopathology domains was significant at a Bonferroni adjusted alpha level of 0.0125 (Z = −4.027. *p* < 0.001). Therefore, supporting hypothesis one, the ANN model performed significantly better than the linear model at predicting lower-level psychopathology.

The bivariate correlations between each higher-level psychopathology factor scores and the linear model and ANN predicted scores are presented in Table 4.

Once again, for each of the three higher-level symptom domains the ANN model’s predicted values had a stronger correlation with the actual values when compared to the linear model. The correlations between the linear model’s predicted values and the actual symptom values ranged between 0.421 and 0.488, while the correlations between the ANN models’ predicted values and the actual symptom values ranged between 0.619 and 0.666. The difference between the linear and ANN models’ correlations with the actual values for internalising, externalising, and the *p*-factor was significant at a Bonferroni adjusted alpha level of 0.0125. The ANN model was more accurate than the linear model at predicting internalising (Z = −3.679. *p* < 0.001), externalising (Z = −3.867. *p* < 0.001), and *p*-factor scores (Z = −3.842. *p* < 0.001). Therefore, supporting hypothesis two, the ANN model performed significantly better than the linear model at predicting lower-level psychopathology.

## 5. Discussion

The aim of this research is to compare the accuracy of linear models versus non-linear artificial neural network models with regard to how well they each predict (a) lower-level and (b) higher-level psychopathology. Overall, we found support for non-linear interactive relationships between the neurocognitive predictors and psychopathology. The ANN models were significantly more accurate than the linear models at predicting both lower-level and higher-level psychopathology. There is consensus that there is a high level of heterogeneity of neurocognition within psychopathology [4,9], however understanding of the variability has been limited primarily by the use of descriptive or linear approaches and the use of DSM diagnostic categories. Previously, through computational modelling, we found that multiple different executive functioning profiles were able to account for the general neurocognitive performance of people with schizophrenia [8]. This finding provided initial support for the multidimensional hypothesis, however, was limited by using a DSM defined disorder category that ignores that dimensionality and comorbidity of psychopathology. Using a dimensional approach, we find that the non-linear multidimensional conceptualisation is superior to traditional linear conceptualisations of the associations and functionality between neurocognition and psychopathology. Given that it is claimed that neurocognition an aetiological feature of psychopathology [1,2], an accurate functional conceptualisation is fundamental to improving our understanding of psychopathology.

Previously, the search for a primary deficit of neurocognition within psychopathology has dominated the literature [24]. While an understanding of a general trend of dysfunction across a specific population may be useful as a starting point to fuller understanding, our findings suggest further assessment of the within individual functionality of neurocognition is required. To illustrate, we recently found that measures of speed of processing, but not working memory, shifting, or inhibition, could significantly account for higher-level psychopathology linearly [23]. However, as in the present research the ANN models were superior in accuracy to the linear models, it suggests that working memory, shifting, and/or inhibition likely still play an important role in understanding the associations between neurocognition and psychopathology. Ultimately, as per the multidimensional hypothesis, the interactions between neurocognitive processes seem integral to a detailed understanding of the associations and functionality between neurocognition and psychopathology.

The use of dimensional, rather than categorical, conceptualisations of psychopathology in the present research has multiple strengths, including mitigating or accounting for the issues of comorbidity and diagnostic stability of the nosological approach [17]. However, examining the multidimensionality of neurocognition with regard to statistically derived higher-level factors of psychopathology does have conceptual considerations. While the lower-level scores of dimensional psychopathology (e.g., depression, hostility, etc.) were not factorised, scores of higher-level factors of psychopathology are intrinsically influenced by the scores of the population from which they were derived. For example, Lahey, Moore, Kaczkurkin and Zald [19] suggests that the *p*-factor is a “weighted average” (p. 61) of the sample’s symptoms. Therefore, the *p*-factor (and internalising and externalising) scores on the individual level are dependent on the factors loadings of the indicators included in the sample model. Indeed, we have previously found that the underlying weightings of different lower-level psychopathology domains vary considerably between different samples [22]. Findings such as these have led to the understanding that higher-level psychopathology factors may not have a universal substantive meaning [22,38]. Considering the substantive interpretation difficulties of higher-level psychopathology, lower-level dimensional psychopathology may be better suited to enhance our understanding the dynamics of neurocognition and psychopathology on the individual level.

An individual approach to neurocognition within developmental conditions, such as intellectual disability and autism spectrum disorder, is common in case conceptualisations and treatment approaches [48,49]. However, even although neurocognitive deficits are highly prevalent, albeit to generally a lesser severity, in psychopathology, this level of assessment and understanding is not commonplace [50]. Our findings indicate that the multidimensionality, rather than general deficits, of neurocognition may be important to consider when understanding an individual’s psychopathology. Further, our results imply that, beyond just strengths and weakness assessment common amongst developmental conditions’ case conceptualisation, a consideration of the interactions between different neurocognitive domains’ performance on the individual level may be important to understanding a person’s psychological experience.

### Limitations and Directions for Future Research

This research has four primary limitations. First, the data was collected online through Prolific [35]. Therefore, we had little control over the conditions under which data were obtained. However, there is evidence that the quality of task data collected through online platforms, in particular Prolific, is comparable to in-lab data [51,52,53,54]. Second, age and gender were required to be predictors in both the linear and ANN models due to their associations between both neurocognition and psychopathology. While the role of age and gender in the linear models is easy to interpret, due to the structure and function of the ANN models the role age and gender played in these models is difficult to parse. Third, the comparisons between the linear models and the ANN models were able to provide evidence that the multidimensional conceptualisation of neurocognitive abilities in psychopathology is superior to the linear conceptualisation. However, our approach to the assessment of the ANN models was unable to provide the necessary information to detail the nuance of the multidimensional functionality. For example, we were not able to provide results for what neurocognitive profiles existed in the data, the specific interaction functionality, and what, if any, compensatory profiles existed. Nonetheless, the current research establishes the importance of considering multidimensional explanations and provides future research with a platform for which to build upon. Lastly, in this study the type of ANN models we developed were among the more accessible of techniques in their respective domains. More complex regression techniques, as well as more complex machine learning techniques exist. Examining how well some of these more complex techniques compare to one another, remains of interest to us for future work. Related to this last point, we also did not test a range of other architectures, activation functions, or use a larger data set. Though clearly each of these offer possible avenues for further study.

Future research should use tightly controlled lab-based data collection to explore non-linear multidimensional conceptualisations. Future research should also attempt to map the neurocognitive profiles that exist amongst the population, the functional dynamics of the neurocognitive domains, and their associations to dimensional psychopathology. More complex regression techniques and more complex machine learning techniques should be also examined and compared by future research. This knowledge may be used to inform aetiological theories of neurocognition and psychopathology and inform case conceptualisations on the individual level. Future research that uses a combination of computational modelling approaches [8], ANN approaches, and descriptive approaches may extend our knowledge of the non-linear multidimensionality.

## 6. Conclusions

In this research, we examined if neurocognitive ANN models were superior to linear models at predicting dimensional lower-level and higher-level psychopathology. We found support for the non-linear multidimensionality of neurocognition in psychopathology as the ANN models were significantly more accurate than the linear models at predicting both lower-level and higher-level psychopathology. We suggest that a non-linear multidimensional conceptualisation of neurocognition within psychopathology is integral for aetiological examination and case conceptualisations. We also suggest that, due to the difficulties in interpreting the substantive meaning of higher-level factors of psychopathology, the utility of examining the multidimensional functionality of neurocognition and psychopathology is greatest at the lower levels of psychopathology using dimensional measures.

## Figures and Tables

**Table 1:** Lower-Level Psychopathology Linear Model Outcomes.

Dependent Variable	Parameter	B	Β	Sig.
Depression	Age	−0.024	−0.396	<0.001 **
	Gender	0.275	0.137	0.007 **
	Digit Span	−0.051	−0.091	0.067
	Visual WM	0.002	0.014	0.790
	Inferring Relevance	117.25	0.030	0.598
	Shape-Number	155.76	0.046	0.410
	Stroop	−337.60	−0.049	0.334
	Go/NoGo	−512.58	−0.030	0.550
	Simple RT	−2866.43	−0.059	0.250
	IT	0.003	0.068	0.177
Agoraphobia	Age	−0.015	−0.241	<0.001 **
	Gender	0.221	0.111	0.007 **
	Digit Span	−0.002	−0.004	0.913
	Visual WM	−0.002	−0.018	0.673
	Inferring Relevance	−65.82	−0.017	0.714
	Shape-Number	−84.80	−0.025	0.578
	Stroop	100.15	0.014	0.722
	Go/NoGo	−709.85	−0.042	0.305
	Simple RT	−3372.44	−0.070	0.094
	IT	0.002	0.038	0.342
Hostility	Age	−0.015	−0.238	<0.001 **
	Gender	0.036	0.018	0.566
	Digit Span	0.018	0.032	0.306
	Visual WM	0.000	−0.004	0.905
	Inferring Relevance	61.31	0.016	0.659
	Shape-Number	−39.83	−0.012	0.736
	Stroop	96.77	0.014	0.657
	Go/NoGo	−293.64	−0.017	0.584
	Simple RT	−5504.99	−0.114	<0.001 **
	IT	0.002	0.050	0.112
Mental Fog	Age	−0.025	−0.410	<0.001 **
	Gender	0.348	0.174	<0.001 **
	Digit Span	−0.029	−0.051	0.245
	Visual WM	−0.002	−0.017	0.718
	Inferring Relevance	35.30	0.009	0.859
	Shape-Number	−37.12	−0.011	0.825
	Stroop	22.51	0.003	0.942
	Go/NoGo	−1498.37	−0.089	0.050
	Simple RT	−1794.27	−0.037	0.419
	IT	0.004	0.081	0.071
Interpersonal Anxiety	Age	−0.022	−0.358	<0.001 **
	Gender	0.214	0.107	0.007 **
	Digit Span	−0.007	−0.012	0.747
	Visual WM	−0.003	−0.027	0.522
	Inferring Relevance	40.08	0.010	0.817
	Shape-Number	−143.92	−0.043	0.329
	Stroop	−271.37	−0.039	0.319
	Go/NoGo	53.71	0.003	0.936
	Simple RT	−4043.57	−0.083	0.038 *
	IT	0.004	0.081	0.038 *
Somatisation	Age	−0.014	−0.228	<0.001 **
	Gender	0.272	0.136	<0.001 **
	Digit Span	−0.005	−0.008	0.776
	Visual WM	0.000	0.002	0.941
	Inferring Relevance	−89.74	−0.023	0.501
	Shape-Number	107.08	0.032	0.345
	Stroop	−57.52	−0.008	0.783
	Go/NoGo	−766.38	−0.045	0.136
	Simple RT	−5405.81	−0.112	<0.001 **
	IT	0.003	0.070	0.020 *
Alcohol	Age	−0.029	−0.468	0.223
	Gender	−1.62	−0.811	0.022 **
	Digit Span	0.240	0.430	0.214
	Visual WM	−0.041	−0.356	0.343
	Inferring Relevance	−297.26	−0.076	0.848
	Shape-Number	−393.96	−0.117	0.765
	Stroop	−682.54	−0.099	0.779
	Go/NoGo	8663.47	0.513	0.149
	Simple RT	−69,091.17	−10.426	<0.001 **
	IT	0.021	0.461	0.188
Cannabis	Age	−0.052	−0.857	0.005 **
	Gender	−0.646	−0.323	0.252
	Digit Span	−0.183	−0.328	0.234
	Visual WM	0.012	0.106	0.722
	Inferring Relevance	1064.69	0.273	0.389
	Shape-Number	−295.90	−0.088	0.778
	Stroop	861.75	0.124	0.657
	Go/NoGo	4069.70	0.241	0.394
	Simple RT	−34,946.23	−0.721	0.012 *
	IT	0.002	0.046	0.868
Other Substances	Age	0.014	0.223	0.648
	Gender	−0.332	−0.166	0.712
	Digit Span	0.533	0.954	0.031 *
	Visual WM	−0.112	−0.980	0.040 *
	Inferring Relevance	1196.78	0.307	0.545
	Shape-Number	1123.99	0.335	0.503
	Stroop	5111.74	0.738	0.100
	Go/NoGo	475.96	0.028	0.950
	Simple RT	−61,098.64	−1.261	0.006 **
	IT	0.024	0.525	0.238

Note. * is significant at the 0.05 level. ** is significant at the 0.01 level. WM = Working Memory. RT = Reaction Time. IT = Inception Time.

**Table 2:** Higher-Level Psychopathology Linear Model Outcomes.

Predictors	Internalising			Externalising			*p-*Factor		
	*B*	β	*p*	*B*	β	*p*	*B*	β	*p*
Age	−0.027	−0.433	<0.001 **	−0.026	−0.346	<0.001 **	−0.398	−0.434	<0.001 **
Gender	0.360	0.174	<0.001 **	0.007	0.003	0.951	4.68	0.156	0.001 **
Digit Span	−0.024	−0.041	0.360	0.036	0.054	0.249	−0.251	−0.030	0.505
Vis WM	−0.002	−0.013	0.783	−0.003	−0.022	0.669	−0.026	−0.015	0.758
Infer. Rel.	13.03	0.003	0.950	149.99	0.032	0.555	415.57	0.007	0.891
Shape-Num	−9.37	−0.003	0.958	−61.02	−0.015	0.778	−214.33	−0.004	0.934
Stroop	−182.03	−0.025	0.578	264.25	0.031	0.664	−1916.14	−0.018	0.686
Go/NoGo	−840.70	−0.048	0.297	−203.95	−0.010	0.835	−11,229.5	−0.044	0.336
Simple RT	−4973.28	−0.099	0.034 *	−12,291.5	−0.209	<0.001 **	−84,882.8	−0.117	0.012 *
IT	0.004	0.094	0.040 *	0.005	0.082	0.083	0.064	0.095	0.037 *

Note. * is significant at the 0.05 level. ** is significant at the 0.01 level. Vis WM = Visual Working Memory. Infer. Rel. = Inferring Relevance. Shape-Num = Shape-Number. RT = Reaction Time. IT = Inspection Time.

**Table 3:** Correlations Between Predicted and Actual Lower-Level Psychopathology Scores.

	LM-Dep	ANN-Dep	LM-Agor	ANN-Agor	LM-Host	ANN-Host	LM-Fog	ANN-Fog	LM-Int. Anx	ANN-Int. Anx	LM-Soma	ANN-Soma	LM-Alc	ANN-Alc	LM-Cann	ANN-Cann	LM-Other	ANN-Other
Dep	0.435	0.648																
Agor			0.331	0.577														
Host					0.414	0.655												
Fog							0.476	0.711										
Int. Anx									0.449	0.675								
Soma											0.469	0.710						
Alc													0.268	0.338				
Cann															0.235	0.413		
Other																	0.243	0.552

Note. All correlations significant at *p* < 0.01 (one-tailed). LM = Linear Model. ANN = Artificial Neural Network Model. Dep = Depression. Agor = Agoraphobia. Fog = Mental Fog. Int. Anx = Interpersonal Anxiety. Soma = Somatisation. Alc = Alcohol use. Cann = Cannabis Use. Other = Other Substance use.

**Table 4:** Correlations Between Predicted and Actual Higher-Level Psychopathology Scores.

	LM-Int	ANN-Int	LM-Ext	ANN-Ext	LM-*p*	ANN-*p*
Internalising	0.488	0.661				
Externalising			0.421	0.619		
*p-*factor					0.486	0.666

Note. All correlations significant at *p* < 0.01 (one-tailed). LM = Linear Model. ANN = Artificial Neural Network Model. Int = Internalising. Ext -= Externalising. *p* = *p*-factor

## Data Availability

Data may be made available upon request granted that the request accommodates for ethical clearances.
